# Cd(II) and Pd(II) Mixed Ligand Complexes of Dithiocarbamate and Tertiary Phosphine Ligands—Spectroscopic, Anti-Microbial, and Computational Studies

**DOI:** 10.3390/molecules28052305

**Published:** 2023-03-02

**Authors:** Tohama B. Abdullah, Reza Behjatmanesh-Ardakani, Ahmed S. Faihan, Hayfa M. Jirjes, Mortaga M. Abou-Krisha, Tarek A. Yousef, Sayed H. Kenawy, Ahmed S. M. Al-Janabi

**Affiliations:** 1 Department of Chemistry, College of Science, University of Tikrit, Tikrit 34001, Iraq; 2 Department of Chemistry, Payame Noor University, Tehran 19395-4697, Iran; 3 Chemistry Department, Science College, Imam Mohammad Ibn Saud Islamic University, Riyadh 11623, Saudi Arabia; 4 Department of Chemistry, South Valley University, Qena 83523, Egypt; 5 Mansoura Laboratory, Toxic and Narcotic Drug, Forensic Medicine Department, Medicolegal Organization, Ministry of Justice, Cairo 11435, Egypt; 6 Ceramics and Building Materials Department, National Research Centre, Refractories, El-Buhouth St., Dokki, Giza 12622, Egypt

**Keywords:** picolylamine, dithiocarbamate, single-pot reaction, DFT, complexes

## Abstract

Mixed ligand complexes of Pd(II) and Cd(II) with *N*-picolyl-amine dithiocarbamate (PAC-dtc) as primary ligand and tertiary phosphine ligand as secondary ligands have been synthesized and characterized via elemental analysis, molar conductance, NMR (^1^H and ^31^P), and IR techniques. The PAC-dtc ligand displayed in a monodentate fashion via sulfur atom whereas diphosphine ligands coordinated as a bidentate mode to afford a square planner around the Pd(II) ion or tetrahedral around the Cd(II) ion. Except for complexes [Cd(PAC-dtc)_2_(dppe)] and [Cd(PAC-dtc)_2_(PPh_3_)_2_], the prepared complexes showed significant antimicrobial activity when evaluated against Staphylococcus aureus, Pseudomonas aeruginosa, Candida albicans and Aspergillus niger. Moreover, DFT calculations were performed to investigate three complexes {[Pd(PAC-dtc)_2_(dppe)](1), [Cd(PAC-dtc)_2_(dppe)](2), [Cd(PAC-dtc)_2_(PPh_3_)_2_](7)}, and their quantum parameters were evaluated using the Gaussian 09 program at the B3LYP/Lanl2dz theoretical level. The optimized structures of the three complexes were square planar and tetrahedral geometry. The calculated bond lengths and bond angles showed a slightly distorted tetrahedral geometry for [Cd(PAC-dtc)_2_(dppe)](2) compared to [Cd(PAC-dtc)_2_(PPh_3_)_2_](7) due to the ring constrain in the dppe ligand. Moreover, the [Pd(PAC-dtc)_2_(dppe)](1) complex showed higher stability compared to **Cd(2)** and **Cd(7)** complexes which can be attributed to the higher back-donation of Pd(1) complex.

## 1. Introduction

**Figure 1:**
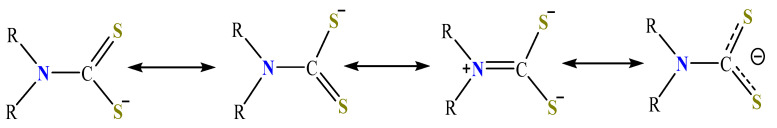
Dithiocarbamate resonance structures.

Growing interest in ligands bearing N and S donor atoms have attracted a lot of attention due to their interesting structural and biological properties [1,2,3,4,5,6,7,8]. Dithiocarbamate, a unique dithiolate ligand, has been increasingly reported in the literature because of its strong chelating capability [2]. This behavior can be explained in terms of its stable resonance structures (Figure 1).

**Figure 2:**
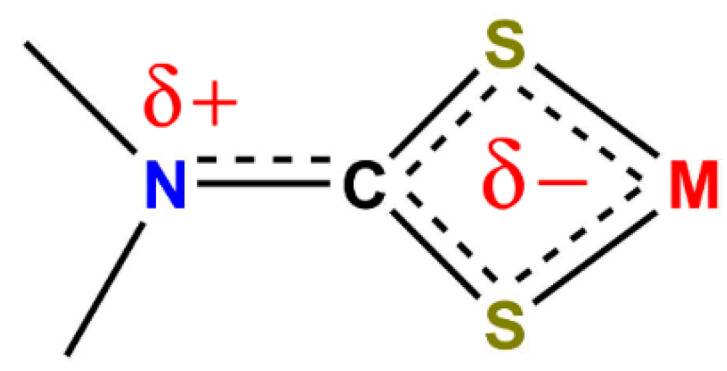
π-electrons delocalization through N-CS_2_ group.

The resulting delocalized pi-electrons through the N-CS_2_ group can provide additional stability (Figure 2).

In addition, the d π vacant orbital on the S atom can serve as a multi donor atom with multiple pi-bonds [9]. Despite the vast literature coverage of dithiocarbamate metal complexes, interest has only been directed at the secondary amine dithiocarbamate. Primary amine dithiocarbamate on the other hand, has been poorly represented in the literature [10]. The acidic proton accompanied with its low stability could be the reason primary amines are not a good candidate for dithiocarbamate synthesis [10]. Cadmium dithiocarbamate complexes have received little attention and only a few examples have been shown in the literature [11,12,13]. The majority of the reported complexes showed dimer characteristics. Cadmium with a dithiocarbamate moiety has also been shown to coordinate with donor atoms of sulfur, nitrogen, and phosphorus [14]. However, the dithiocarbamate ligands and their complexes have drawn considerable interest because of their intriguing biological characteristics [15,16,17]. Moreover, they have applications in areas as diverse as medicine, agriculture, biological imaging, and materials science; they are extremely versatile ligands, forming stable complexes with all the transition metals, along with the lanthanides, the actinides, and a majority of the p-block elements. At the same time, their ability to adopt resonance hybrids allows for stabilizing metals in both high and low oxidation states [16,17,18,19,20,21,22,23,24,25,26]. Over the past decade, the development of dithiocarbamate chemistry has continued unabated with applications in areas including novel inorganic drugs, capping agents for nano-engineered surfaces and other nanomaterials, including quantum dots, and the rational construction of well-defined complex molecular structures [17,18,19,20,21,22,23,24,25,26].

In the current efforts to develop the coordination chemistry of primary amine di-thiocarbamate complexes, including phosphine ligands, we used the *N*-picolyl-amine dithiocarbamate metal complex (MII = Cd, Pd) as a base for the synthesis of some mixed ligand complexes containing tertiary phosphine ligands.

## 2. Results

### 2.1. Synthesis

**Scheme 1:**
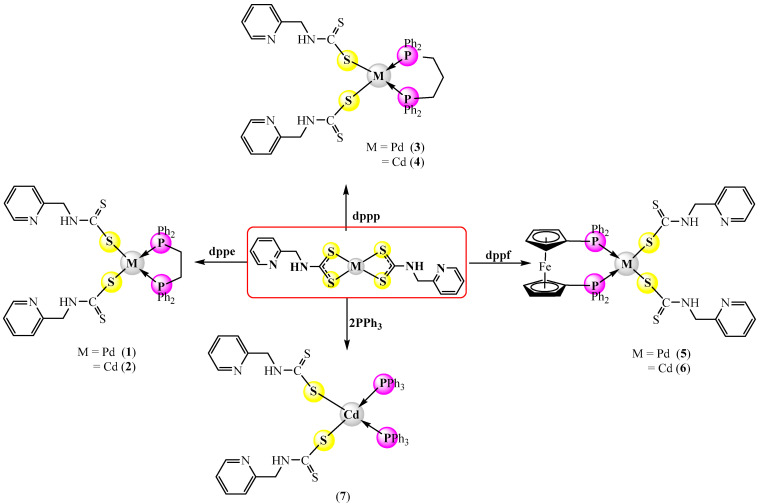
Synthesis of complexes (**1**–**7**).

The heteroleptic Pd(II) and Cd(II) complexes were synthesized by treating [Pd(PCA-dtc)_2_] and [Cd(PCA-dtc)_2_] with equivalent molar diphosphine (dppe, dppp, dppf) or with two equivalents of triphenyl phosphine (PPh_3_) under reflux conditions in chloroform (Scheme 1). They are stable in air, and their solubility was found in DMSO and DMF and was analyzed by elemental analysis, molar conductivity and spectroscopic techniques (^1^H, ^13^C NMR, mass, IR, and UV-visible). The molar conductivity of the complexes in DMSO revealed that they were neutral as expected [27]. The results indicated that PAC-dtc ligands were bonded as a monodentate fashion through the sulfur atom, whereas the diphosphine ligands (dppe, dppp and dppf) bonded as bidentate chelating, while triphenyl phosphine was coordinated as monodentate model. The geometry around Pd(II) ion is a square plane, but a tetrahedral around Cd(II) ion.

### 2.2. Characterization of Prepared Complexes

Characterization was relatively natural on the fundamental of spectral and analytical data (Table 1, Table 2 and Table 3).

#### 2.2.1. ^31^P-{^1^H} and ^1^H nmr Spectra

The ^31^P-{^1^H} nmr spectra of the complexes (**1**–**7**) showed a singlet peak for each at δ 30.42 ppm, δ 37.19, δ 30.42 ppm, δ 37.19, δ 30.42 ppm, δ 37.19 ppm, and δ 30.22 ppm, respectively (Figures S1–S7) and (Table 2), indicating the appearance of a single isomer for each and the phosphorus atoms are equivalents; we propose that this isomer is maybe the S-bonded one.

In the ^1^H NMR spectra of complexes (**1**) and (**2**) (Figures S8 and S9), they displayed a singlet peak at δH 2.43 and 2.72 ppm for CH_2_ of dppe ligand, respectively. Whereas the CH_2_ of **PAC-dtc** ligand was displayed at δH 4.93 and 4.40 ppm, respectively. In addition, the spectra showed the proton of the NH group at δH 11.58 and 13.37 ppm, respectively. Finally, the phenyl protons of the diphosphine and **PAC-dtc** ligands appeared as unresolved multiplets within the δ 6.63–8.84 ppm range. Integration of the signals agreed well with the formula. The ^1^H NMR spectra of complexes (**3**) and (**4**) (Figures S10 and S11), displayed two broad singlets at δH (3.01, 3.09) ppm and δH (1.89, 1.84) ppm corresponding to the two different methylene groups for the phosphine ligand. The protons for the CH_2_ and NH groups of **PAC-dtc** ligand appeared as two separated peaks at δH (4.32, 4.46) and δ (10.28, 11.03), respectively. The phenyl protons of the phosphine and PAC-dtc ligands appeared as multiplets within the δH 6.81–8.39 ppm range.

In ^1^H NMR spectra of complexes (**5**) and (**6**) (Figures S12 and S13), the CH_2_ and NH groups of **PAC-dtc** ligand appeared as two separate peaks at δH (4.21, 4.35) ppm and δH (10.71, 10.84) ppm, respectively, whereas the protons of the cyclopentadiene of dppf ligand appeared at δH 4.84 ppm, for the two complexes, respectively. The phenyl protons showed as unresolved multiplet peaks within the δH 6.70–8.85 ppm range. The ^1^H NMR spectrum of [Cd(PCA-dtc)_2_(PPh_3_)_2_] (**7**) (Figure S14), showed two peaks at δH 4.25 and 10.45 ppm due to the CH_2_ and NH groups for the **PAC-dtc** ligand, respectively. The phenyl protons were showed as unresolved multiplets peak within the δH 6.64–8.68 ppm range.

#### 2.2.2. IR Data

The IR data of the synthesized complexes were assigned and are presented in Table 3. Three new bands displayed in the IR spectra of the [M(PAC-dtc)_2_(diphos)] {M^II^ = Pd and Cd} complexes which were not found in the spectra of [M(PAC-dtc)_2_] complexes, within (1433–1437) cm^−1^, (1093–1120) cm^−1^ and (497–532) cm^−1^ range, due to the υ(P-Ph), υ(P-C)_stretching_, and bending of (P-C) band, respectively [28,29,30,31,32,33,34]. It is thought [34] that this vibration arises from the deformation of the planarity of the phenyl ring bonded to a heavy atom (phosphorus).

The CS_2_ thiocarbonyl stretching splits into two peaks (double and single) with medium intensity within (694–700) cm^−1^ and (1036–987) cm^−1^ range for complexes (**1**–**7**) [35,36]. The spectroscopic data suggest that the PAC-dtc ligand coordinated in a monodentate fashion through the sulfur atom with Pd(II) and Cd(II)ions. In addition, the IR spectra displayed the NH vibrations within the (3291–3338) cm^−1^ range. Whereas the frequencies C=N of pyridyl ring and C–N aliphatic appeared within (1595–1624) cm^−1^ and (1531–1548) cm^−1^. Two other new characteristic bands appeared within (412–440) cm^−1^ and (462–497) cm^−1^ assigned to the υ(M-S) [36,37,38] and υ(M-P) [36,37], respectively.

### 2.3. Antimicrobial Studies

Seven mixed ligand complexes (**1**–**7**) were screened for their antibacterial activity against *Staphylococcus aureus* and *Pseudomonas aeruginosa* using the cup-plate method [33]. Additionally, the antifungal ability of these complexes was tested against two fungal species *Candida albicans* and *Aspergillus niger* using the disc diffusion method [39]. The standard error for the experiment was ±0.03%, and the experiments were repeated three times in the same conditions.

The antibacterial activity results against the tested bacteria revealed that most of the compounds have shown moderate to excellent activity. Complexes **5** and **6** displayed good antibacterial activity against pathogenic bacteria species in comparison with the standard Gentamicin and other prepared compounds, which may be due to the presence of iron element in the structure of diphosphine ligand (dppf) in addition to the electron-donating atoms (N and S) of pyridyl and dithiocarbamate groups. The antibacterial activity order is as follows:

**5** > **6** > **1** > **4** > **3** > **2** > **7**

In similar antifungal results, it is evident that Complexes **5** and **6** displayed good to excellent restraint effects against *Candida albicans* and *Aspergillus niger* compared to Fluconazole. It may be due to the presence of iron element in the structure of dppf ligand with electron-donating atoms (N and S) of pyridyl and dithiocarbamate groups; the results of these antimicrobial activities are listed in Table S1.

### 2.4. Computational Studies

#### 2.4.1. Geometrical and Electronic Properties

**Figure 3:**
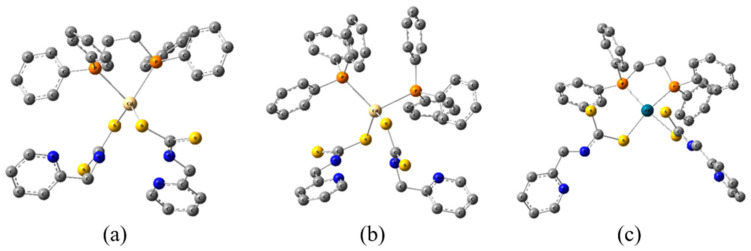
Optimized structures for (**a**) **Cd(2)**, (**b**) **Cd(7)** and (**c**) **Pd(1)**; hydrogen atoms were not shown for simplicity.

The optimized structures of complexes (**Pd(1)**, **Cd(2)**, **Cd(7)**) are shown in (Figure 3). These complexes all have a (**+2**) central ion due to the hydrogen loss of SH in each S-containing ligand. Two structures—square planar and tetrahedral geometry—have been found in the geometry of the complexes under study. Pd(II) in the **Pd(1)** complex, for instance, has square planar geometry, but Cd(II) in the **Cd(2)** and **Cd(7)** complexes has tetrahedral geometry. The calculated bond angles have shown different values for each complex. For instance, Cd(**2**) and Cd(**7**) have substantially different angles between Cd(II) and the donor atoms. The bond angle of P-Cd-P in Cd(**2**) is 79.5°, while it is 109.2° in Cd(**7**). In addition, the S-Cd-S angle in Cd(**2**) and Cd(**7**) is 122.5° and 120.9°, respectively. These results demonstrate that the distortion in Cd(**2**) is greater than the distortion in Cd(**7**). Tetrahedral symmetry in its optimal form has an angle of 109.5°. However, the angles in the Cd(**2**) and Cd(**7**) complexes are not perfect. **Pd(1)** complex one the other hand, showed a distorted square planar geometry. The calculated bond angles are 100, 86, 87 and 87°, which are normal for square planar configuration. Similarly, the bond lengths values of the complexes under study have also shown some variations. These differences arise from the fact that the dppe ligand has generated a constraint on the overall complex structure which affects both the bond distances and the bond angles. For instance, the Cd-S bond lengths of Cd(**2**) are 2.50 and 2.52 Å, while in Cd(**7**) complex these are 2.52 and 2.54 Å. The Cd-P bond lengths in Cd(**2**) complex are 2.74 and 2.77 Å, whereas in Cd(**7**) complex these are 2.77 and 2.84 Å.

**Figure 4:**
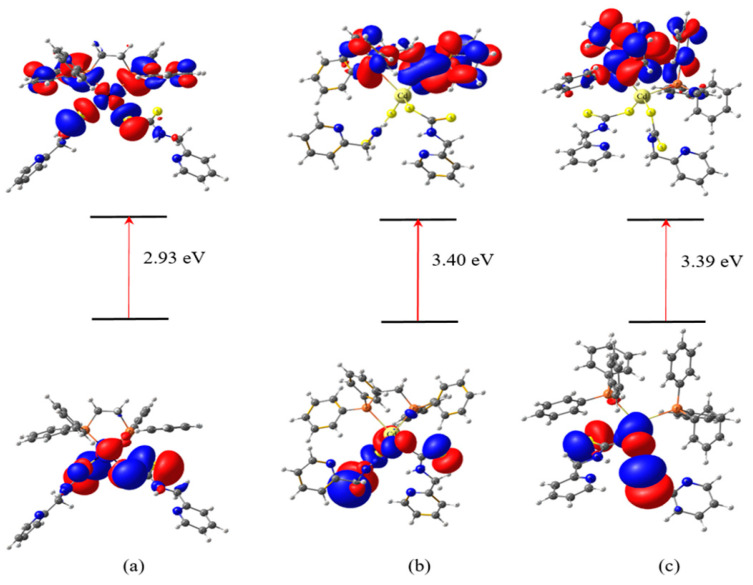
HOMO and LUMO for (**a**) Pd(1), (**b**) Cd(2) and (**c**) Cd(7).

The highest occupied molecular orbital (HOMO) and the lowest unoccupied molecular orbital (LUMO) are calculated at the level of B3LYP/Def2-TZVP (Figure 4). The HOMO-LUMO energy gap for **Pd(1)** is less than the two other complexes. These findings imply that Pd(**1**) is a good candidate for a photo catalyst material that activates electron-hole pairs in the solar energy zone. According to Figure 4, HOMO are primarily localized on ligands that contain S, while LUMO are primarily localized on ligands that contain P. This is consistent with the estimated charges on the atoms of P and S. (see Table 4). The electronegativity of P atoms is lower than that of S and N atoms. In contrast to their role in LUMO, the P atoms do not contribute to HOMO charge density.

#### 2.4.2. NBO Analysis

In Table 5, the natural bond orbital (NBO) charges for selected atoms in the complexes under study have been listed. The core metal ions’ charges in each of the three complexes shift from +2 to a less positive value. These outcomes show a charge transfer from ligand to metal. The NBO charge for Cd changes from +2 to +1.26, while the NBO charge for Pd changes from +2 to +0.26. This shows that there is a higher ligand to metal charge transfer (LMCT) for Pd compared to Cd. The NBO charges on S atoms are negative, but less than one, which indicates that these atoms donate their valence charge to the Cd(II)/Pd(II) atoms. The results demonstrate that S atom in **Pd(1)** complex has a lower negative charge than S atoms in **Cd(2)** and **Cd(7)** complexes, indicating a stronger electron donation to Pd (II). However, compared to two other complexes, the NBO charge on the P atom in the **Pd(1)** complex is greater positive. As a result, the **Pd(1)** complex had a stronger charge donation from ligand to metal than the **Cd(2)** and **Cd(7)** complexes.

**Figure 5:**
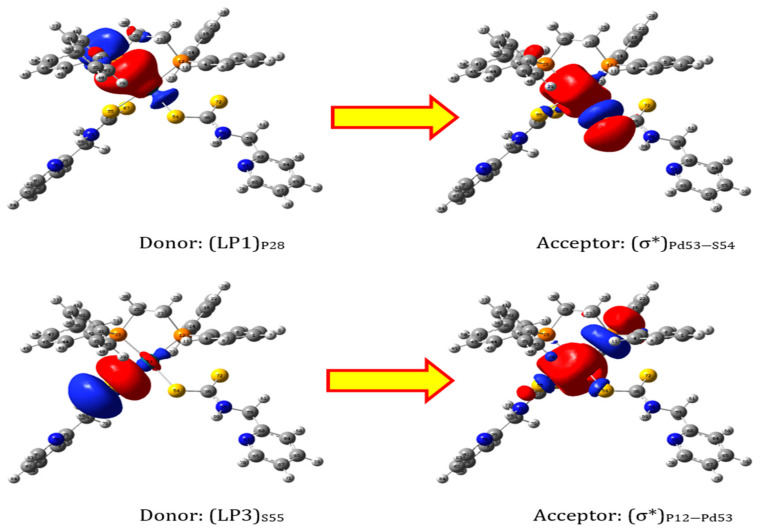
Donor and acceptor orbitals of two highest second order perturbation energies of **Pd(1)** complex.

**Figure 6:**
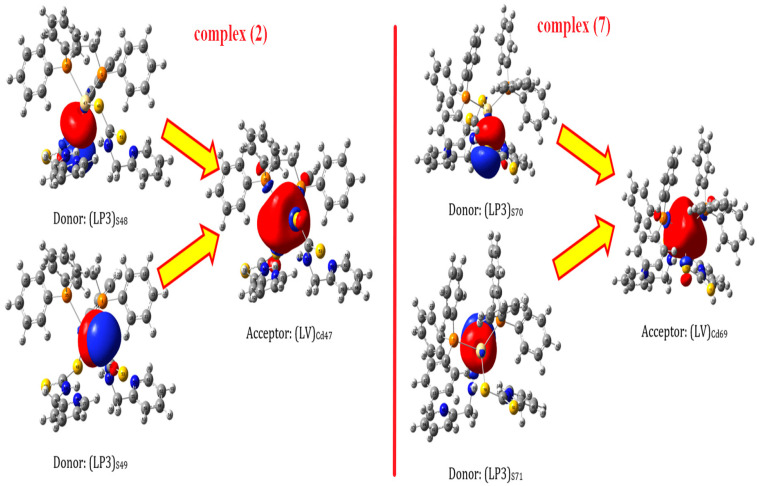
Two highest *E*^(2)^ donors and acceptor orbitals for **Cd(2)** and **Cd(7)** complexes.

Second order perturbation energy (*E*^(2)^) was calculated to investigate how much these charge transfers stabilize the complexes. Table 5, Table 6 and Table 7 show the results of the second order perturbation energies due to the charge transfer from donor orbitals to the acceptor ones. Figure 5 and Figure 6 graphically represent donor and acceptor orbitals for the two highest second order perturbation energies. In line with the previous discussion, P and S atoms are very good electron donors to the central metal ions. The maximum *E*^(2)^ for **Cd(2)** and **Cd(7)** complexes belong to S donors, while the highest *E*^(2)^ for **Pd(1)** complex belong to P donors. P orbitals (in hybridized or pure forms) are the main type of donor orbitals in all three complexes. N is a lot less active donor in these complexes compared to our earlier study [16]. Additionally, unlike the Pt complexes examined earlier [37], there is no back-donation charge transfer in these complexes. Based on the obtained data, the 6s orbital in **Cd(2)** and **Cd(7)** complexes is the best electron acceptor. According to NBO analysis, **Pd(1)** complex’s anti-binding orbitals of Pd-S and Pd-P are the best electron acceptors.

#### 2.4.3. MEP Analysis

**Figure 7:**
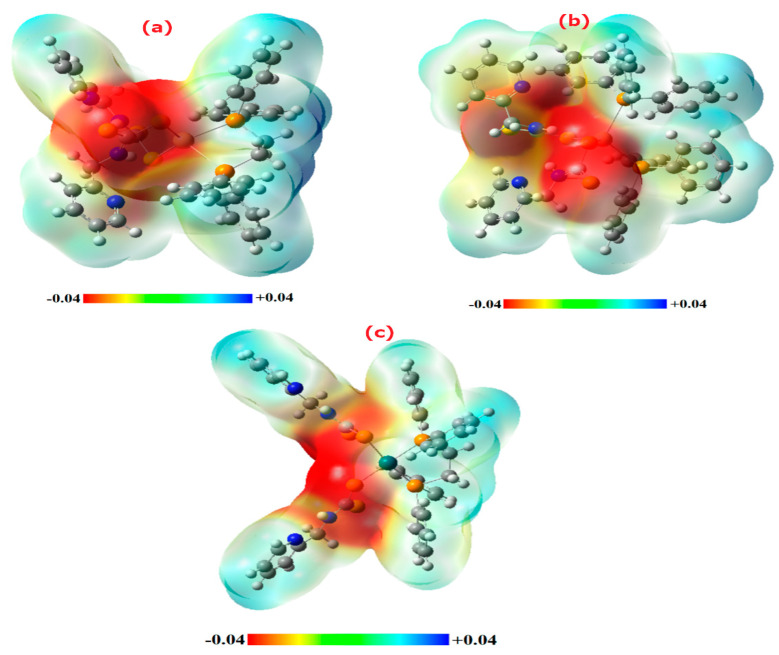
Molecular electrostatic potential calculated at the level of B3LYP/Def2-TZVP for (**a**) **Cd(2)** (**b**) **Cd(2)**, and (**c**) **Pd(1)**.

From a formatted check-point file, the examined complexes’ molecular electrostatic potential was estimated. To do this, total electronic density was considered for MEP calculations. Different colors are used to depict the charge distribution. The red color is for negative MEP showing electron rich regions, and the blue color is for positive MEP that shows regions of electron deficiency. The highest positive and negative values of MEP were fixed to be in the range of −0.04 to +0.04 (Figure 7).

## 3. Experimental Part

### 3.1. Material and Physical Measurements

The NMR and IR spectra were recorded using an NMR-Bruker spectrophotometer (400 MHz- in DMSO-d_6_ solvent) and FT-IR 8400 spectrophotometer (as KBr disc in 4000–400 cm^−1^ range). Elemental (CHN) analysis was recorded by Elemetar Vario EL Cube. The molar conductivity is measured using (JENWEAY-Molar Conductivity meter) for a DMSO solution of the prepared complexes with 10^−3^ M at 25 °C. All materials used in this study were purchased from Merck or Alfa-Aser and used without further purification. The [Pd(PCA-dtc)_2_] and [Pd(PCA-dtc)_2_] complexes were according to the literature [40].

### 3.2. Synthesis of Complexes

#### 3.2.1. Synthesis of [Pd(PCA-dtc)_2_(dppe)]Complex (**1**)

A solution of 1,2-bis(diphenylphosphino)ethane (dppe) (0.025 g, 0.063 mmol) in CHCl_3_ (10mL) was added to a solution of a complex [Pd(PCA-dtc)_2_] (0.030 g, 0.063 mmol) in CHCl_3_ (10 mL), and a yellow suspension was formed. The mixture was refluxed for four hours. The greenish yellow mixture was left at room temperature to evaporate slowly, then a gum matter was formed, washed with ether and n-hexane several times to afford a greenish yellow ppt. then dried under vacuum. (Greenish yellow solid powder. Yield: 0.050 g, 91%. M.p (°C): 190–193).

The following complexes [Cd(PCA-dtc)_2_(dppe)] (**2**), [Pd(PCA-dtc)_2_(dppp)] (**3**), [Cd(PCA-dtc)_2_(dppp)] (**4**), [Pd(PCA-dtc)_2_(dppf)] (**5**), [Cd(PCA-dtc)_2_(dppf)] (**6**) were prepared and isolated in similar methods.

#### 3.2.2. Synthesis of [Cd(PCA-dtc)_2_(PPh_3_)_2_]complex (**7**)

A solution of triphenyl phosphine (PPh_3_) (0.049 g, 0.191 mmol) in CHCl_3_ (10 mL) was added to a solution of a complex [Cd(PCA-dtc)_2_] (0.022 g, 0.045 mmol) in CHCl_3_ (10mL), and a yellow suspension was formed. The mixture was refluxed for four hours. The greenish yellow mixture was left at room temperature to evaporate slowly, then a gum matter was formed, washed with ether and n-hexane several times to afford a greenish yellow ppt. then dried under vacuum. (Green solid powder, Yield: 0.044 g, 96%. M.p (°C): 101–103).

### 3.3. Antibacterial Studies

The biological activity of metal-ligand complexes (**1**–**7**) were examined as antibacterial activity against *Staphylococcus aureus* and *Pseudomonas aeruginosa*. Additionally, the antifungal ability of these complexes was tested against two fungal species *Candida albicans* and *Aspergillus niger* using the disc diffusion method using the cup-plate method at 10^−3^ M concentration of its and the result was compared with Gentamicin for anti-bacterial and or Fluconazole for anti-fungi as positive drug [39]. The standard error for the experiment was ±0.03%, and the experiments were repeated three times in the same conditions.

### 3.4. Computational Detail

Three different complexes were selected to study their electronic properties. At first, the structures of these complexes (**Pd(1)**, **Cd(2)**, **Cd(7)**) were optimized with B3LYP/Def2-SVP level of theory by using the Gaussian 09 program [41]. To be sure that these optimized structures are in the local minima points, the frequency analyses were performed. One negative frequency shows that the structure is in the transition state, two or more negative frequencies show that the structure is in the saddle point, and zero negative frequency shows that the structure is in the local minimum point in the potential energy. After genuine structure optimization, post-processing calculations were performed to determine the examined complexes’ electrical characteristics. Frontier orbitals (**HOMO** and **LUMO**) and electrostatic potential (**ESP**) were calculated with B3LYP/Def2-TZVP level of theory. They were calculated from a formatted checkpoint file at the iso-surface density value of 0.02 e/${Å}^{3}$. Natural bond orbital (**NBO**) calculations such as natural charges and donor-acceptor stabilization energy were created with the NBO 6.0 program [42] at B3LYP/Def2-SVPD level. The GaussView program [43] was used to show contour plot of HOMO and LUMO.

## 4. Conclusions

In this study, we describe the synthesis, characterization and in-vitro antibacterial, and cytotoxic activity of novel mixed ligand Pd(II) and Cd(II) of *N*-picolyl-amine dithiocarbamate and phosphine ligands. The dithiocarbamate ligand coordinate as monodentate through sulfur atom. The tested compounds were examined against *Staphylococcus aureus*, *Pseudomonas aeruginosa*, *Candida albicans* and *Aspergillus niger.* Complexes **5** and **6** displayed good antibacterial activity against pathogenic bacteria and fungi species in comparison with the standard drugs and other prepared compounds. Three complexes **{Pd(1)**, **Cd(2)**, **Cd(7)}** were theoretically investigated via the DFT method. The obtained data supported the spectroscopic charts at which these complexes adopt square planar and tetrahedral geometries. However, the tetrahedral geometry was not perfect and a distorted tetrahedral structure was exhibited due to the effect of the dppe ligand.

## Figures and Tables

**Table 1:** Physical properties and elemental analysis.

Seq.	Color	Yield (%)	Conductivity	m.p (°C)	Elemental Analysis Calc. (Found) %		
					C	H	N
1	Greenish yellow	91	12.1	190–192	55.14 (54.93)	4.40 (4.61)	6.43 (6.78)
2	White	74	8.0	162–165	54.76 (54.90)	4.37 (4.20)	6.39 (6.45)
3	Greenish yellow	89	3.8	152–154	55.62 (55.78)	4.55 (4.72)	6.33 (6.51)
4	Olive	86	2.9	89–92	55.24 (55.37)	4.52 (4.43)	6.29 (6.48)
5	Dark brown	92	10.1	168–174	56.12 (56.06)	4.12 (3.98)	5.45 (5.71)
6	Olive	45	11.0	121–124	55.79 (55.81)	4.10 (3.96)	5.42 (5.70)
7	Green	96	13.8	101–103	59.84 (59.74)	4.42 (4.71)	5.58 (5.62)

**Table 2:** ^31^P and ^1^H nmr chemical shifts of the synthesized complexes.

Complexes	δP (ppm)	δH (ppm)			
		NH	CH_2_ or H-Cp _PAC-dtc_	CH_2 phosphine_	Aryl Rings
1	30.60	11.58 (*s*, 2H)	4.93 (*s*, 4H)	2.43 (*s*, 4H)	7.49–8.84 (*m*, 28H)
2	30.34	13.37 (*bs*, 2H)	4.40 (*s*, 4H)	2.72 (*s*, 4H)	6.63–8.11 (*m*, 28H)
3	30.23	10.28 (*s*, 2H)	4.32 (*s*, 2H)	3.01 (*bs*, 4H); 1.89 (*bs*, 2H);	6.97–8.11 (*m*, 28H)
4	30.28	11.04 (*bs*, 2H)	4.46 (*s*, 2H)	3.09 (*s*, 4H); 1.84 (*s*, 2H);	6.81–8.39 (*m*, 28H)
5	26.00	10.71 (*s*, 2H)	4.35 (*s*, 4H)	4.84 (*bs*, 8H)	6.96–8.85 (*m*, 28H)
6	39.83	10.84 (*d*, 2H)	4.21 (*d*, 4H)	4.90–5.28 (*m*, 8H)	6.70–8.66 (*m*, 28H)
7	25.83	10.45 (*d*, 2H)	4.25 (*dd*, 4H)	--	6.64–8.68 (*m*, 38H)

**Table 3:** IR data of the synthesized complexes in (cm^−1^).

Comps.	ν(N-H)	ν(C-H)		Phosphine Ligands			ν(C=N)_pyridyl_ ν(C-N)	ν(CS_2_)	ν(M-P) ν(M-S)
		Aliph.	Arom.	ν(P-Ph)	ν(P-C)_st_	δ(P-C)			
1	3203b	3057w	2943w	1435s	1119s	532s	1618m 1533s	1001m 694s	493w 428w
2	3331m	3008w	2918w	1433s	1093s	530s	1595m 1533s	987m 694s	479w 425w
3	3308b	3108w	2943w	1433s	1120s	511s	1624m 1533s	1001m 694s	463w 424w
4	3338m	3053w	2935w	1437s	1118s	509s	1618m 1541s	995m 694s	464w 427w
5	3318m	3103w	2947w	1433s	1118s	532s	1622s 1531s	1001m 698s	491w 440w
6	3291m	3057w	2953w	1437s	1120s	497s	1623m 1541s	1036m 700m	497w 424w
7	3318m	3053w	2947w	1437s	1120s	502s	1595s 1548s	997m 694m	462w 412w

**Table 4:** NBO charges for the central ion and the connected atoms.

Complex	S	S	P	P	Cd/Pd
Cd(2)	−0.46	−0.41	+0.73	+0.73	+1.27
Cd(7)	−0.47	−0.42	+0.78	+0.78	+1.26
Pd(1)	−0.17	−0.15	+1.0	+1.0	+0.26

**Table 5:** Second order perturbation energies for charge transfers from donor to acceptor orbitals in **Pd(1)**.

Donor	Type	Acceptor	Type	*E* ^(2)^
(LP1)_P28_	13% (*s*) + 87% (*p*)	P12-Pd53	σ *	71.5
(LP1)_P28_	13% (*s*) + 87% (*p*)	C25-P28	σ *	23.6
(LP1)_P28_	13% (*s*) + 87% (*p*)	P28-C31	σ *	22.0
(LP1)_P28_	13% (*s*) + 87% (*p*)	P28-C42	σ *	23.8
(LP1)_P28_	13% (*s*) + 87% (*p*)	Pd53-S54	σ *	236.5
(LP2)_S54_	100% (*p*)	C56-S72	π *	47.0
(LP1)_N58_	100% (*p*)	C56-S72	π *	81.5
)LP2(_S72_	100% (*p*)	C56-N58	σ *	12.1
P12-Pd53	σ	Pd53-S54	σ *	31.5
Pd53-S54	σ	P12-Pd53	σ *	66.7
Pd53-S54	σ	Pd53-S54	σ *	21.5
(LP2(_S55_	74% (*s*) + 26% (*p*)	P12-Pd53	σ *	12.3
(LP3(_S55_	100% (*p*)	P12-Pd53	σ *	106.5
(LP3(_S55_	100%(*p*)	Pd53-S54	σ *	19.4
(LP2(_S55_	100%(*p*)	C57-S87	σ *	43.9
(LP1(_N73_	100%(*p*)	C57-S87	σ *	85.2
(LP2(_S87_	100%(*p*)	C57-N73	σ *	12.5

**Table 6:** Second order perturbation energies for charge transfers from donor to acceptor orbitals in **Cd(2)**.

Donor	Type	Acceptor	Type	*E* ^(2)^
(LP1)_P1_	45% (*s*) + 55% (*p*)	(LV)_Cd47_	100% (*s*)	64.0
(LP1)_S48_	70% (*s*) + 30% (*p*)	(LV)_Cd47_	100% (*s*)	7.4
(LP2)_S48_	7% (*s*) + 93% (*p*)	(LV)_Cd47_	100% (*s*)	7.9
(LP3)_S48_	8% (*s*) + 92% (*p*)	(LV)_Cd47_	100% (*s*)	131.4
(LP3)_S48_	8% (*s*) + 92% (*p*)	C50-S69	π *	22.7
(LP1)_N68_	100% (*p*)	C50-S69	π *	70.8
(LP2)_S48_	7% (*s*) + 93% (*p*)	N52-H67	σ *	8.3
(LP3)_S49_	12% (*s*) + 88% (*p*)	(LV)_Cd47_	100% (*s*)	140.0
(LP2)_S49_	100% (*p*)	C51-S53	σ *	13.0
(LP1)_N52_	100% (*p*)	C51-S53	σ *	24.7
(LP1)_N52_	100% (*p*)	C51-S53	π *	14.8
(LP2)_S53_	100% (*p*)	S49-C51	σ *	10.5
(LP2)_S53_	100% (*p*)	C51-N52	σ *	11.4

**Table 7:** Second order perturbation energies for charge transfers from donor to acceptor orbitals in **Cd(7)**.

Donor	Type	Acceptor	Type	*E* ^(2)^
(LP1)_P7_	45% (*s*) + 55% (*p*)	(LV)_Cd69_	100% (*s*)	58.8
(LP1)_P46_	45% (*s*) + 55% (*p*)	(LV)_Cd69_	100% (*s*)	70.0
(LP3)_S70_	8% (*s*) + 92% (*p*)	(LV)_Cd69_	100% (*s*)	124.3
(LP3)_S70_	8% (*s*) + 92% (*p*)	C72-S91	π *	23.5
(LP1)_N90_	100% (*s*)	C72-S91	π *	72.7
(LP2)_S91_	1% (*s*) + 99% (*p*)	S70-C72	σ *	11.3
(LP2)_S91_	1% (*s*) + 99% (*p*)	C72-N90	σ *	11.4
(LP2)_S70_	8% (*s*) + 92% (*p*)	N74-H89	σ *	10.1
(LP3)_S71_	12% (*s*) + 88% (*p*)	(LV)_Cd69_	100% (*s*)	128.6
(LP2)_S71_	100% (*p*)	C73-S75	π *	41.3
(LP1)_N74_	100% (*p*)	C73-S75	π *	88.5
(LP2)_S75_	100% (*p*)	S71-C73	σ *	10.4
(LP2)_S75_	100% (*p*)	C73-N74	σ *	11.2

## Data Availability

In the supporting information of this article, you will find the data that supports the findings of this study.

## Supplementary Materials

The following supporting information can be downloaded at: https://www.mdpi.com/article/10.3390/molecules28052305/s1, Figure S1: ^31^P nmr spectrum of complex (1), Figure S2: ^31^P nmr spectrum of complex (2), Figure S3: ^31^P nmr spectrum of complex (3), Figure S4: ^31^P nmr spectrum of complex (4), Figure S5: ^31^P nmr spectrum of complex (5), Figure S6: ^31^P nmr spectrum of complex (6), Figure S7: ^31^P nmr spectrum of complex (7), Figure S8: ^1^H nmr spectrum of complex (1), Figure S9: ^1^H nmr spectrum of complex (2), Figure S10: ^1^H nmr spectrum of complex (3), Figure S11: ^1^H nmr spectrum of complex (5), Figure S12: ^1^H nmr spectrum of complex (6) and Figure S13: ^1^H nmr spectrum of complex (7).
