# Supplementary material for: Cd(II) and Pd(II) Mixed Ligand Complexes of Dithiocarbamate and Tertiary Phosphine Ligands—Spectroscopic, Anti-Microbial, and Computational Studies
Source: Molecules. 2023 Mar 2;28(5):2305. doi: 10.3390/molecules28052305 (PMC10005262; doi:10.3390/molecules28052305)

Cd(II) and Pd(II) Mixed Ligand Complexes of Dithiocarbamate and Tertiary Phosphine Ligands—Spectroscopic, Anti-Microbial, and Computational Studies

Tohama B. Abdullah , Reza Behjatmanesh-Ardakani, Ahmed S. Faihan, Hayfa M. Jirjes, Mortaga M. Abou-Krisha, Tarek A. Yousef, Sayed H. Kenawy and Ahmed S. M. Al-Janabi

Figure S1. ^{31}P nmr spectrum of complex (1)

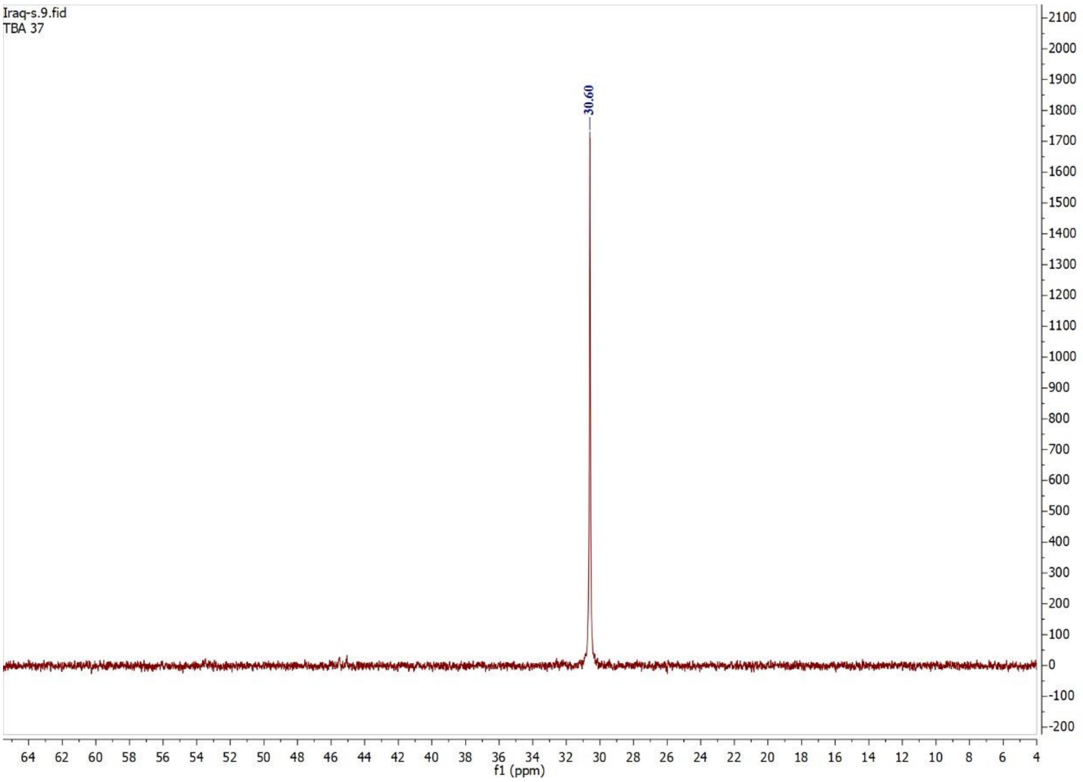

Figure S2. ^{31}P nmr spectrum of complex (2)

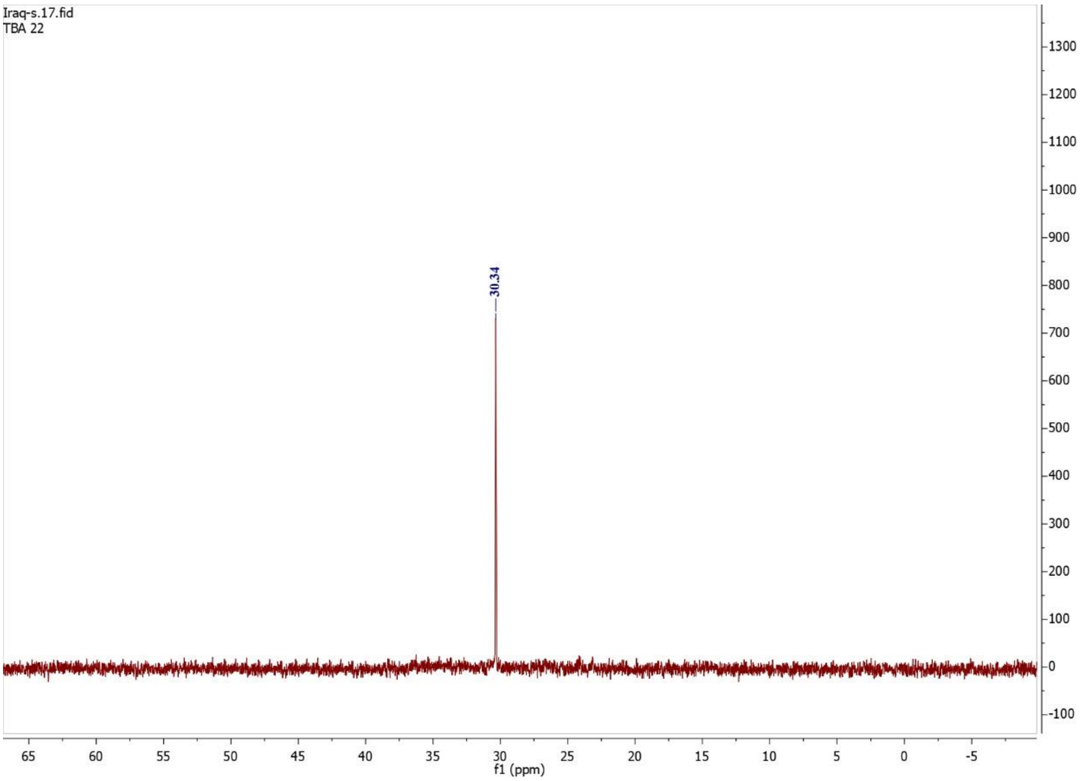

Figure S3. ^{31}**P nmr spectrum of complex (3)**

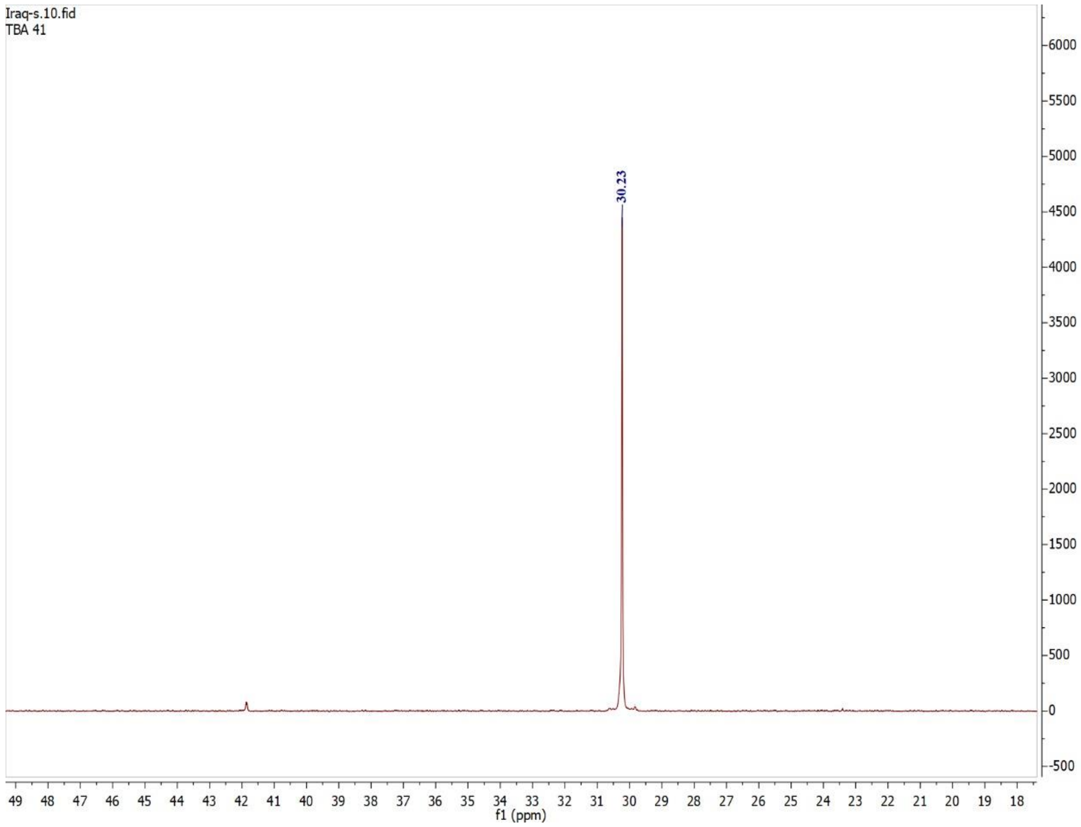

Figure S4. ^{31}**P nmr spectrum of complex (4)**

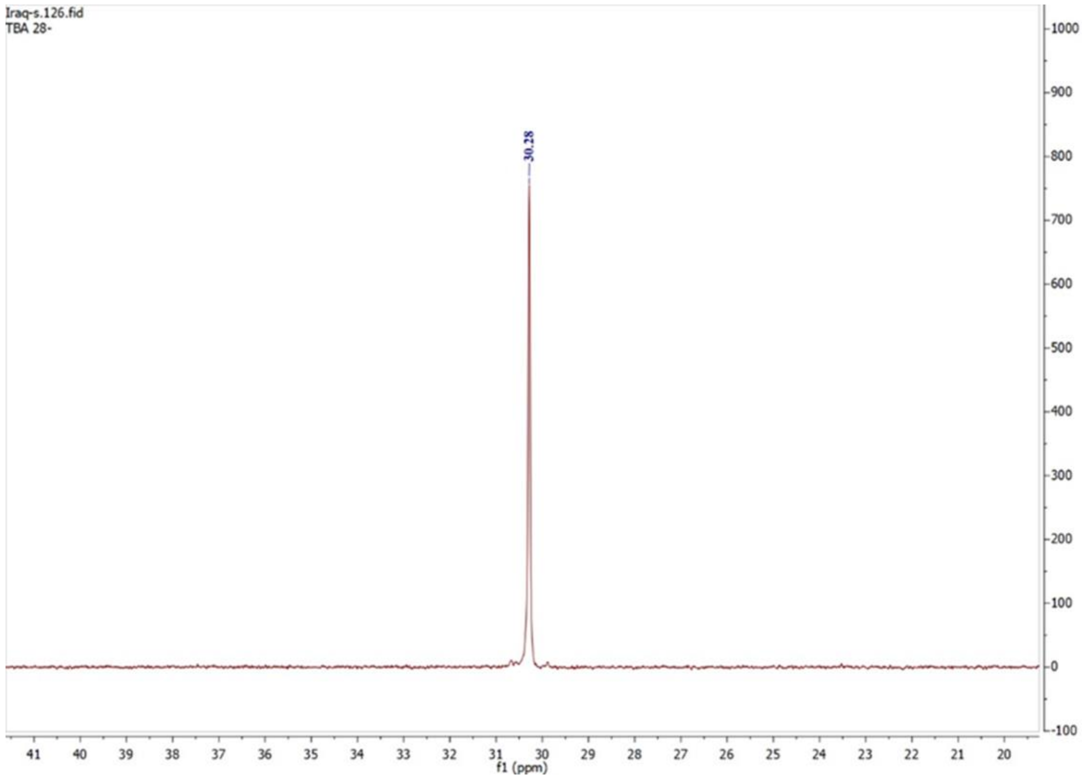

Figure S5. ^{31}P **nmr spectrum of complex (5)**

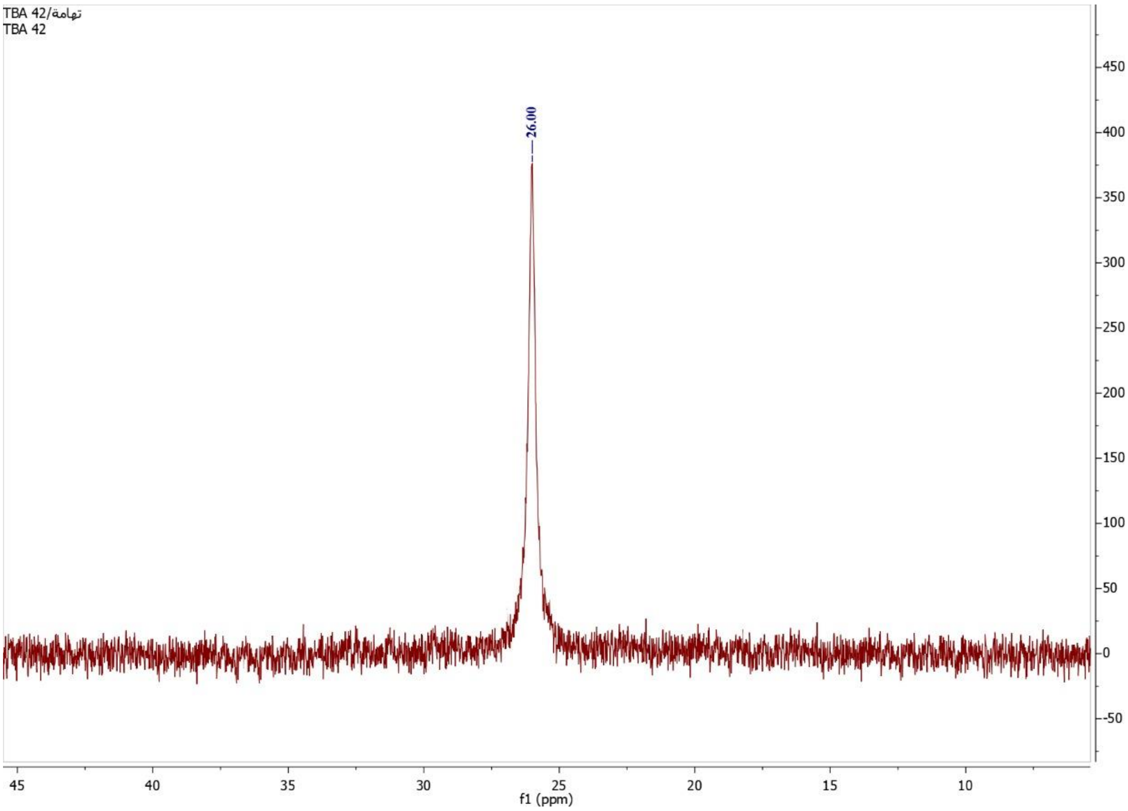

Figure S6. ^{31}P **nmr spectrum of complex (6)**

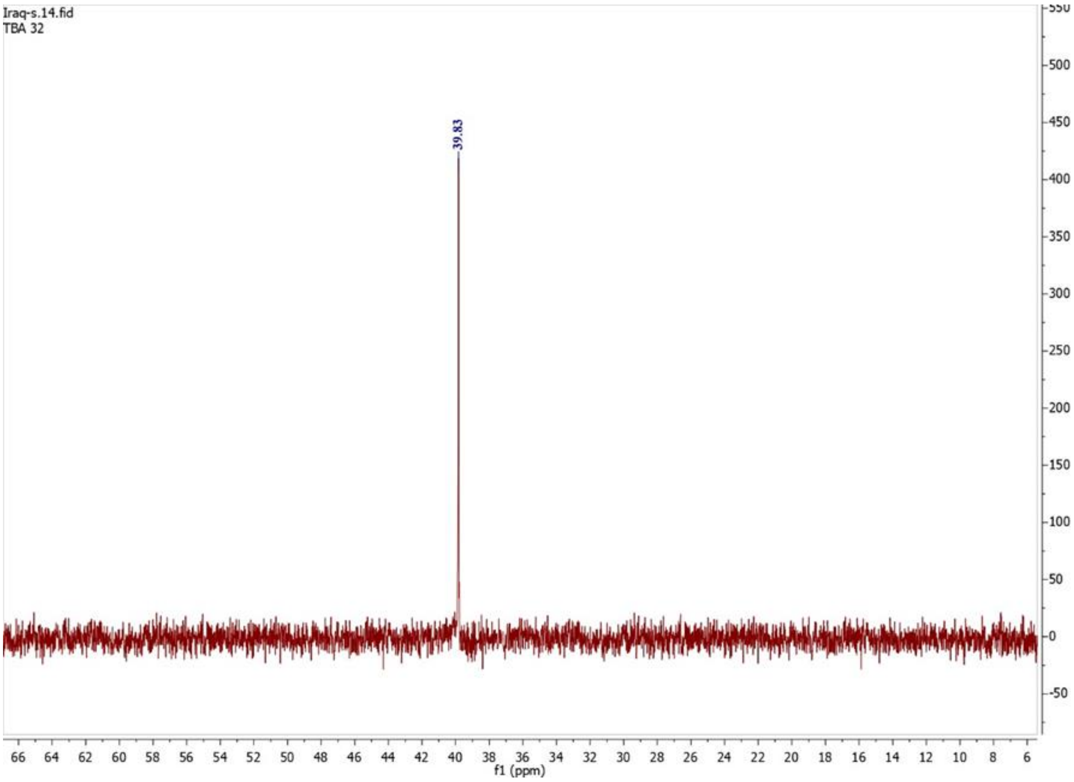

Figure S7. ^{31}P **nmr spectrum of complex (7)**

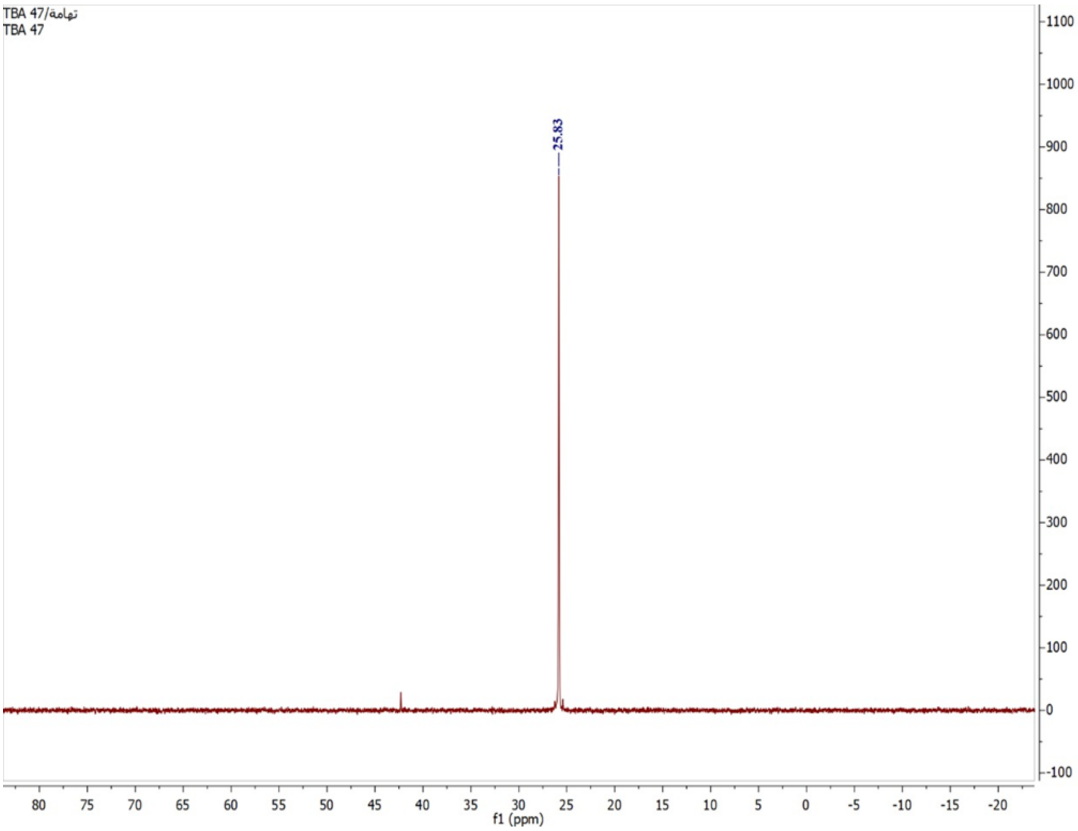

Figure S8. ^{1}H **nmr spectrum of complex (1)**

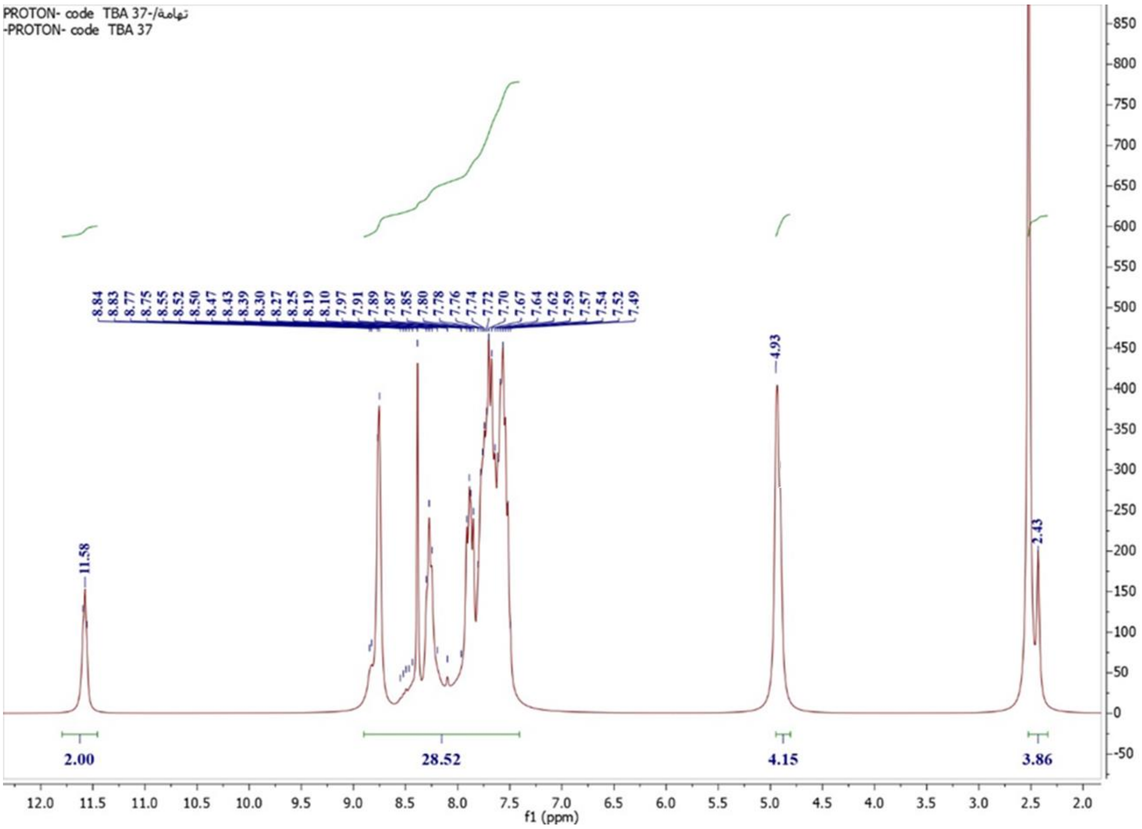

Figure S9. ^{1}H **nmr spectrum of complex (2)**

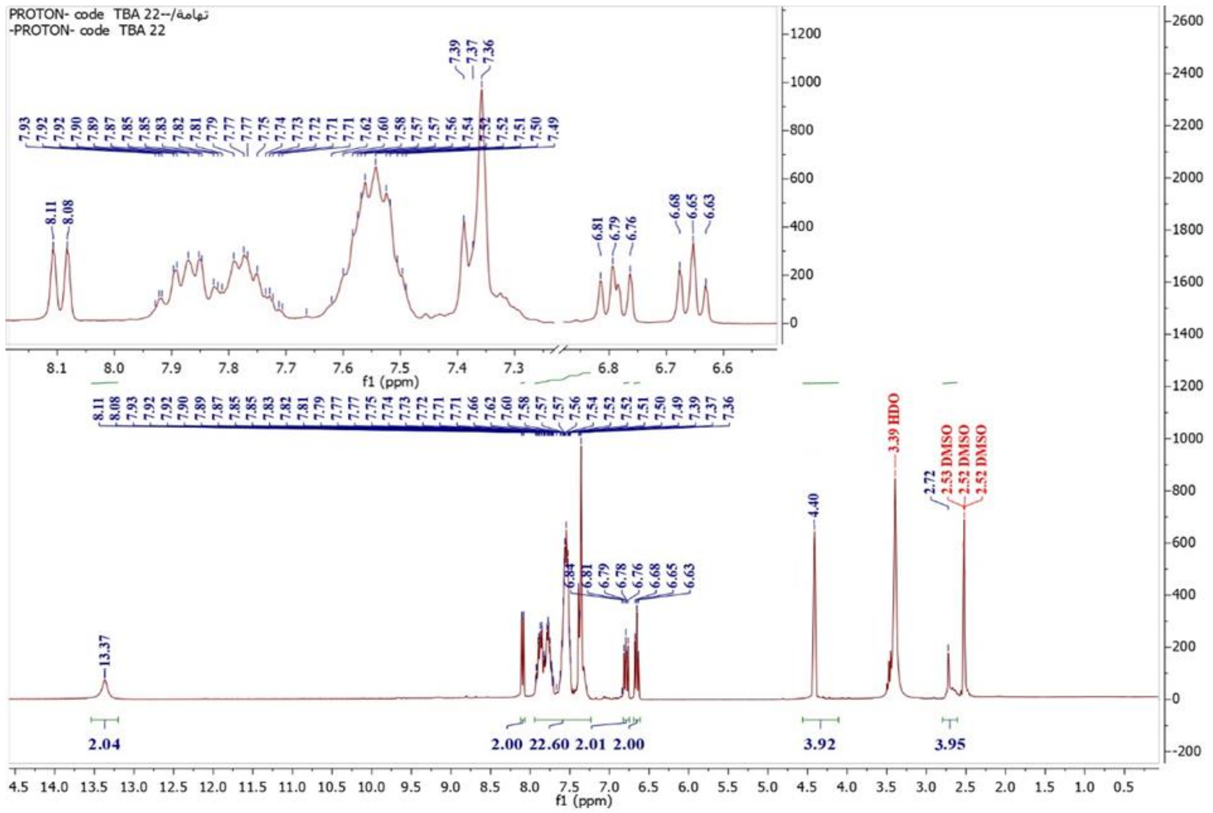

Figure S0. ^{1}H **nmr spectrum of complex (3)**

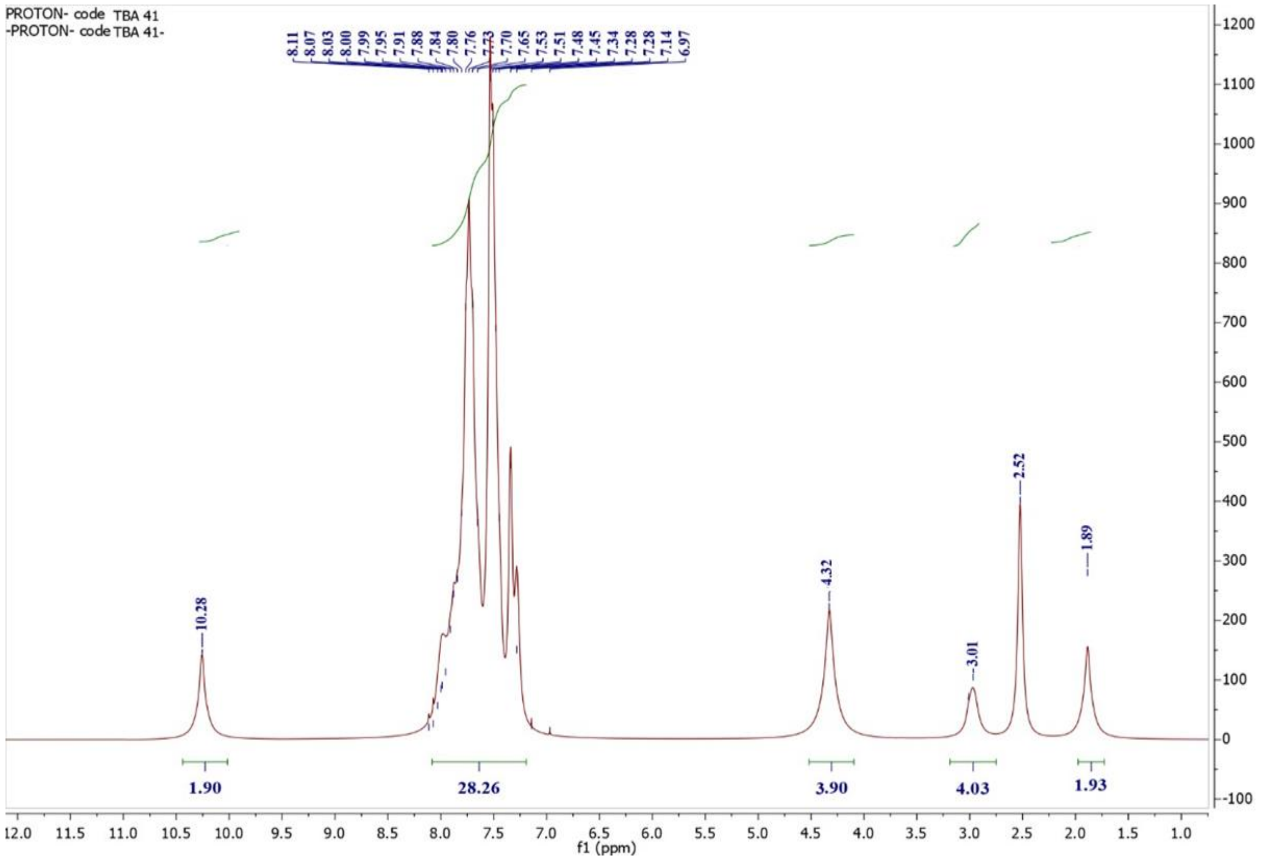

Figure S11. ^{1}H nmr spectrum of complex (5)

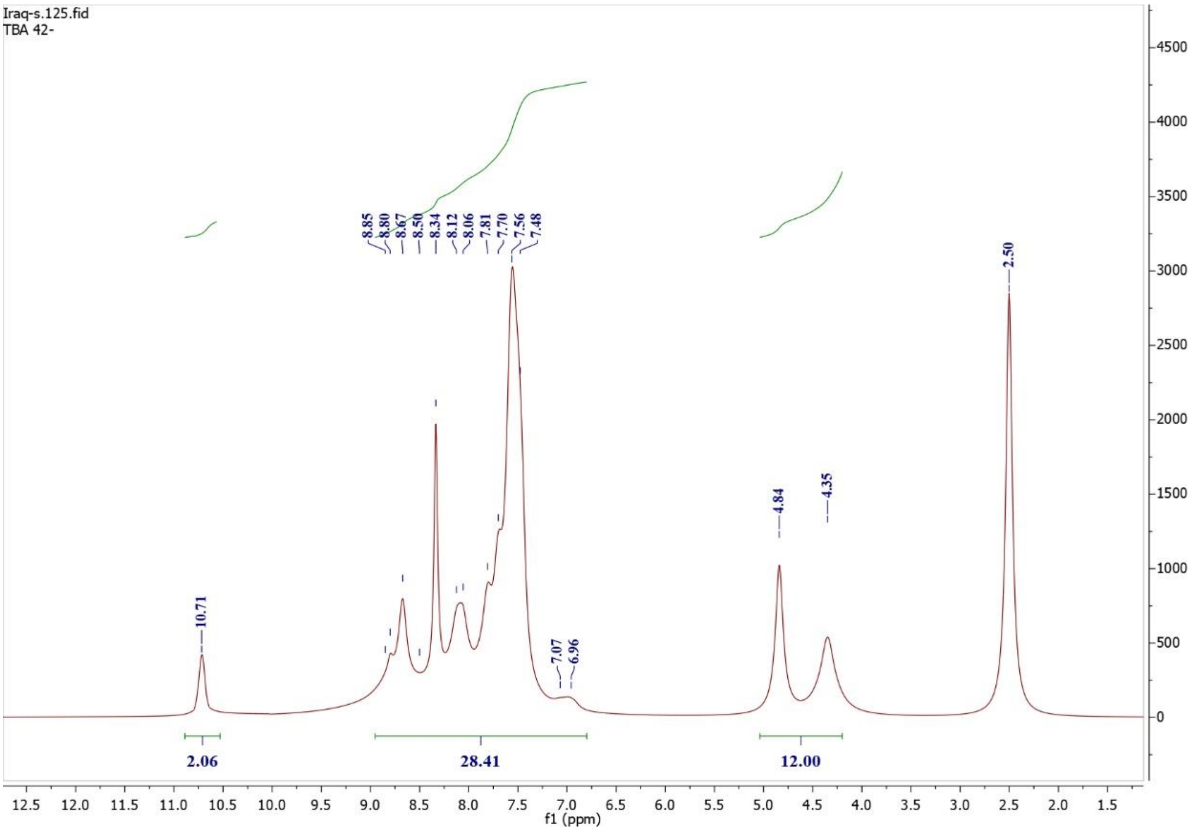

Figure S12. ^{1}H nmr spectrum of complex (6)

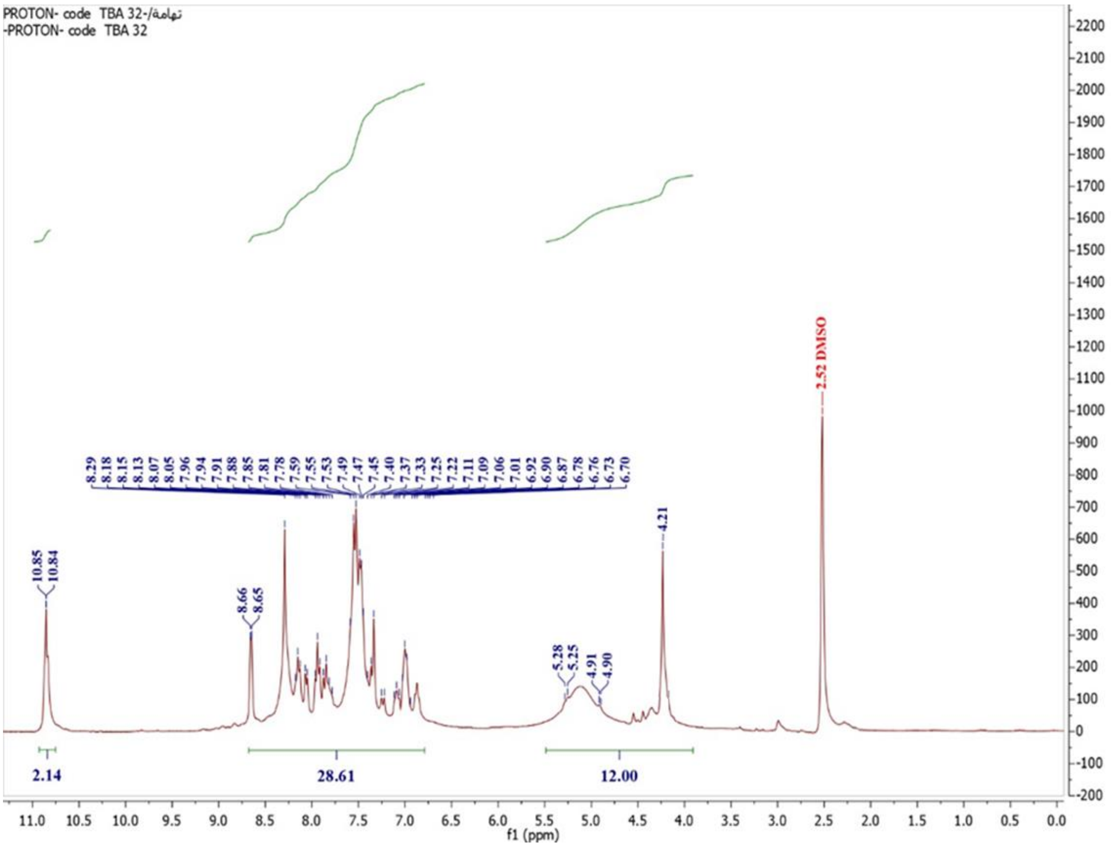

Figure S13. ^{1}H nmr spectrum of complex (7)

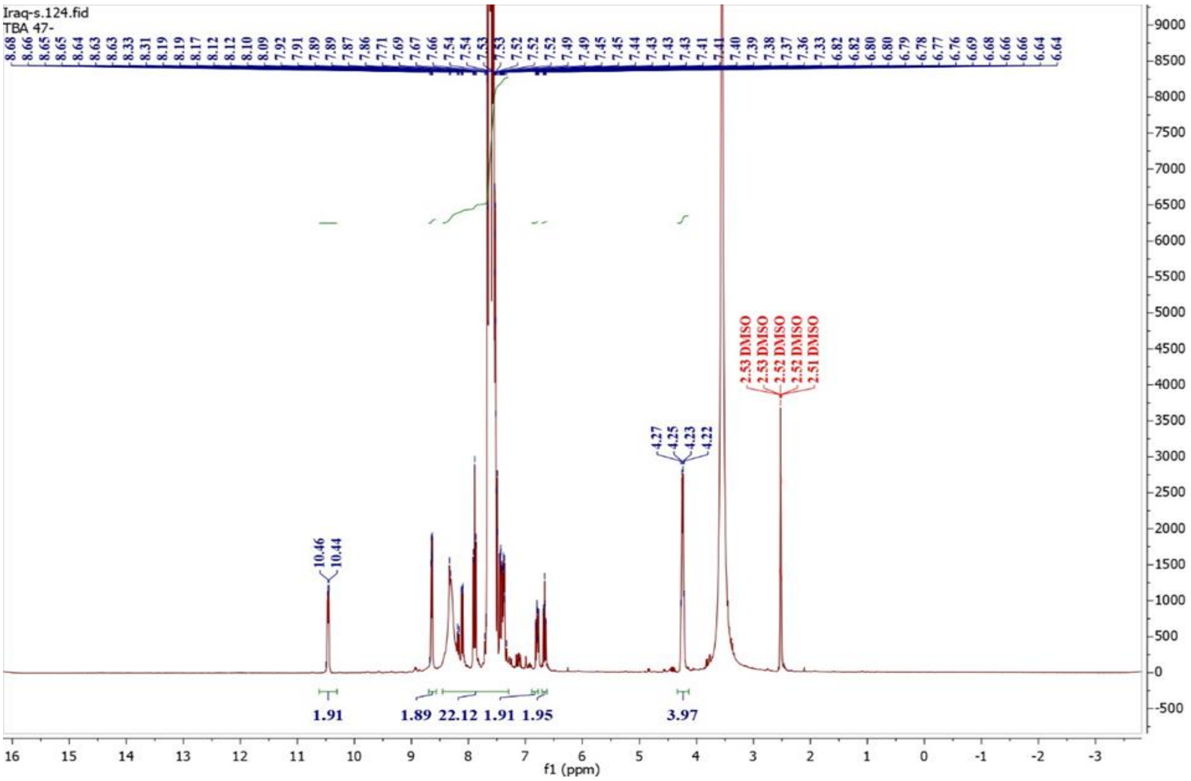

Supplement: Supplementary file 1 [file molecules-28-02305-s001.zip › molecules-2213876-supplementary.pdf]
